# The impact of sewage sludge processing on the safety of its use

**DOI:** 10.1038/s41598-022-16354-5

**Published:** 2022-07-18

**Authors:** Katarzyna Styszko, Justyna Durak, Beata Kończak, Marcin Głodniok, Anna Borgulat

**Affiliations:** 1 Department of Coal Chemistry and Environmental Sciences, Faculty of Energy and Fuels, AGH University of Science and Technology, Al. Mickiewicza 30, 30-059 Kraków, Poland; 2 Department of Geoengineering and Water Management, Faculty of Environmental Engineering and Energy, Cracow University of Technology, Kraków, Poland; 3 Department of Water Protection, Central Mining Institute, Plac Gwarków 1, 40-166 Katowice, Poland

**Keywords:** Environmental chemistry, Environmental impact

## Abstract

Particular attention is devoted to pharmaceutical residues in sewage sludge caused by their potential ecotoxicological effects. Diclofenac, ibuprofen and carbamazepine, 17-α-ethinylestradiol, β-estradiol, were analysed in four types of fertilizers, based on sewage sludge commercial products, in compliance with Polish requirements. The release of active pharmaceutical compounds from fertilizers to water the phase after 24 h and 27 days was analysed. Solid-water partition coefficients (K_d_) and partitioning coefficient values normalized on organic carbon content (log K_OC_) were evaluated. The environmental risk to terrestrial ecosystems, due to the application of fertilizers onto soils, was estimated. Cumulative mass of pharmaceuticals emitted to water from fertilizers ranged from 0.4 to 30.8 µg/kg after 24 h contact. The greatest amount of the material that was released, over 70%, was observed for carbamazepine. No presence of compounds except ibuprofen was observed after 27 days of testing. The highest environmental risk in fertilizers is due to carbamazepine, risk quotation, RQ = 0.93 and diclofenac RQ = 0.17. The values of risk quotation estimated for soil were below RQ = 0.01. This fact means that no risk to terrestrial ecosystems is expected to occur. The important decrease of the concentrations of active compounds after passing from sewage sludge to fertilizers [and] to fertilized soil could be observed.

## Introduction

Various human activities are sources of pharmaceutical residues which can have a significant impact on human health and the condition of ecosystems. The intensive agriculture, the high expansion of urban areas and the use of fertilizers based on sewage sludge causes the entry of contaminants into the environment. One of the main sources of a wide mixture of compounds is associated with sewage treatment plants (WWTP), from which emerging contaminants are discharged with effluents, directly into surface waters^1–3^. Other –indirect –sources are associated with runoff from solid waste from arable land, urban and rural runoff and accidental spills^4,5^. Pollutants from biosolids used in agriculture can also become part of the food chain^6–8^. Sewage sludge is the most important output product of WWTPs. The reuse and disposal of sewage sludge constitute the most complex problem^9,10^. The amount of sewage sludge produced has been consistently high for the past 10 years^11^. The reasons for such a large amount of sewage sludge can be perceived not only in the increasing amount of waste but also the result of using modern, more effective methods of wastewater treatment, mainly biological methods. The wastewater treatment plants (WWTPs) generate large amounts of biosolids each year and their disposal poses a significant problem for the environment. In many countries the agricultural use of sewage sludge is a commonly employed farming practice. However, there are many concerns for possible risks. PPCPs is a group of unregulated contaminants present in sewage sludge fertilizer and are they arebeing studied in this matrix repeatedly. The factor limiting the industrial use of sewage sludge in agriculture is the presence of sanitary pollution or excessive heavy metal content. In accordance with the Fertilizers and Fertilization Act^12^, the only type of sludge that can be used as an organic or organic-mineral fertilizer is the one which contains the right amount of organic substances, nitrogen, phosphorus and potassium. Pharmaceuticals are an important problem that may affect the limitation of the use of sewage sludge as fertilizer products, which results from the specificity of their activity and the possibilities of immunizing pathogenic microorganisms against the active substances contained in the sludge^2,13–19^. According to the data collected by the Polish Ministry of Health, in cooperation with the World Health Organization Regional Office for Europe, the level of consumption of pharmaceuticals in Poland is one of the highest in Europe^20^. The number of non-prescription pharmaceuticals, especially antitussive, analgesic and anti-inflammatory drugs, increases every year^21^. Research on the presence of ibuprofen in urban wastewater also confirms its very low susceptibility to biological treatment and its significant susceptibility to be deposited in sewage sludge^22^.

Non-steroidal anti-inflammatory drugs (NSAIDs) are a main concern because of their wide use by humans and the largest group of over-the-counter drugs sold worldwide^23,24^. The reason that antibiotics and hormones which have been widely used in human and veterinary medicine are the object of particular attention is their potential role in the development of resistant mechanisms by bacteria and effects on the endocrine system of organisms, respectively^17,23,25,26^. The studies of Wu et al., 2021 have shown that the introduction of pharmaceuticals into soil, e.g. with recycled water increase up to 2.3 and 1.8 in trimethoprim and sulfamethoxazole concentrations at 0–120 cm soil depth and reduce the abundance of soil microorganisms^27^. Decision 2015/495 / EU published a watch list of substances for EU-wide monitoring in the field of water policy, containing 17 organic compounds, known as pollutants of new concern, for which extensive monitoring data is needed^23,28,29^. The watch list includes five pharmaceuticals, diclofenac, macrolide antibiotics, as well as synthetic estrogen 17-α-ethinylestradiol (EE2), as well as natural estrogens, estrone (E1) and 17β-estradiol (E2), among others.

Given the fact that it is not possible to purify wastewater from such contaminants, the problem seems to be of great importance in the context of human health. It is frequently the case that pharmaceuticals affect the human hormonal balance, as is the case with contraceptives^25,30–34^. They pose a threat to the environment due to their higher biological activity, despite the fact that the annual production of the synthetic steroid hormones in contraceptive pills, such as 17α-ethinylestradiol (EE2), is much smaller than NSAIDs.It is in the range of a couple of hundred kilogrammes per year in the EU^35^. These compounds cause estrogenic activity in fish at a concentration of 1–4 ng/L, or lower^35^.

Even before 2000, the first review works appeared in which ecological and analytical aspects of pharmacological pollution of sewage sludge were discussed^36–38^. In addition to the presence of pharmaceutical residues in the surface and groundwater, there were also pharmaceuticals which occurred as contaminants in the drinking water^37,39,40^, which most likely resulted from the difficulties in the treatment of chemically complex pharmaceuticals^41^.

The use of treated wastewater and digested sludge, as well as animal manure are ways of transporting pharmaceuticals of human and veterinary origin to soil, and this leads to the accumulation of these substances^42–45^. In the near future, the increase of the input of pharmaceuticals into soils can be expected due to the need of the reduction of the use of freshwater irrigation and chemical-based fertilizers coupled with the reuse of wastewater treatment by-products. The Food and Agricultural Organisation of the United Nations’ in report entitled “Water for Sustainable Food and Agriculture” recommendated that G20 members should consider to invest in the development of the wastewater treatment at urban areas and its reuse in agriculture in order to increase agricultural productivity and climate resilience of local communities. Living organisms of different ecosystems may be affected by pharmaceuticals either directly or indirectly via food web transfer. Nevertheless, the extent of exposure through this pathway remain unknown for most taxa and ecosystems^6^. Physicochemical properties of pharmaceuticals, as well soils influence on their fate in the environment^46–48^.

Nonsteroidal anti-inflammatory drugs (NSAIDs), diclofenac and ibuprofen, which appear in soils due to the application of municipal wastewater or biosolids may migrate through soils intact or be transformed and reach groundwater^49^.Carbamazepine is claimed to be very persistent in the environment since it is highly recalcitrant to standard biological wastewater treatment processes^45^. Particular attention has been already devoted to the hormones and endocrine disruptors which lead to fish feminization^35^. These pollutants have recently been intensively discussed in terms of their constant emissions to the environment, long-term harm to aquatic ecosystems and human health, as well as their growing production and consumption.

The stabilized sewage sludge from the municipal wastewater treatment plants is increasingly widespread in agricultural use, directly or indirectly. Sewage sludge tends to sorb many potential contaminants from wastewater, such as pesticides, pharmaceuticals and other organic chemical residues. These active compounds are emerging as pollutants, not only due to their potential for ecotoxicological risks but also because of their continuous emission to the environment. Therefore, we studied the effects of different conditions such as time of leaching, technology of production of fertilizers on the mobility of pharmaceutical active compounds from the tested fertilizers. It is necessary to introduce solutions that can help to manage the problem of the emerging pollutants, as well as to enable people to reuse sewage sludge in a way that is in line with a circular economy. The technology for granulated fertilizer production from municipal sewage sludge (Polish patent No.233754 from 2019) is to be discussed. The novelty of this technology consists in the mechanical dynamic mixing for formulation granules from sewage sludge and mineral powders. Heretofore this type of granulation has not been used in reference to sewage sludge. By applying this technology it is also possible to use less quicklime due to full homogenization. Therefore the reactive substrates are used more effectively.

In this work, the leaching of selected micro pollutants, 17-α-ethinylestradiol, β-estradiol, carbamazepine, diclofenac and ibuprofen from the sewage sludge fertilizer obtained in different processing methods was tested. The main purpose of the research was to assess the safety of the use of fertilizer products made from processed sewage sludge in terms of releasing pharmacological contaminants. Fertilizer products were subjected to the release process according to the PN-EN-13266 standard. These water extracts were examined after 24 h and 27 days.

## Materials and methods

### Materials

The chemical structures, the physical and chemical properties of 17-α-ethinylestradiol, β-estradiol, carbamazepine, diclofenac, and ibuprofen, were presented in Table A1. 17-α-ethinylestradiol (> 99%), β-estradiol (> 99%), carbamazepine (> 99%), ibuprofen (> 99%), were purchased from Sigma-Aldrich (St. Louis, USA), while diclofenac (99%) was purchased from Dr. Ehrenstorfer (Augsburg, Germany). Internal standards (see Table A1) were purchased from Sigma Aldrich (Saint Louis, USA). Methanol was used in the preparation of solutions of compounds, both singly and as mixtures. For the preparation of a mixture of standard solution, a stock standard solution of 1000 μg/mLof each compound was used. The water and solid samples were spiked with mixtures. For the derivatization, a reagent, N,O-bis(trimethylsilyl)trifluoroacetamide (BSTFA) containing 1% of trimethylchlorosilane (TMCS),(Sigma-Aldrich),was used. GC grade methanol was obtained from Avantor (Gliwice, Poland). Deionized water (< 0.07 S/cm), used to the experiments, was obtained from a HLP5 pure water system (Hydrolab, Gdańsk, Poland).

**Table 1:** Ecotoxicological data of the pharmaceutically active compounds obtained for fertilizers. The Values of RQ_soil_ were much below 0.01 and they are not represented in the table.

Compound	IBF	DCF	CBZ
**PNEC**_**water**_^a^ µg/L	1.65	9.70	13.8
**PNEC**_**soil**_^a^ µg/kg	256	1595	347
**PNEC**_**fertilizer**_ µg/kg (in this study)			
Fertilizer 1	453	1526	264
Fertilizer 2	296	2428	80
Fertilizer 3	2184	10,223	-
Fertilizer 4	195	282	22
**RQ**_**fertilizer**_			
Fertilizer 1	0.02	0.03	0.06
Fertilizer 2	0.03	0.02	0.20
Fertilizer 3	0.00	0.00	-
Fertilizer 4	0.05	0.17	0.93

^a^Journal of Hazardous Materials 239–240 (2012) 40–47.

### Characterization of sewage sludge

The sewage sludge from which the fertilizer was obtained originated from the Gigablok sewage treatment plant, owned by the Katowicka Infrastruktura Wodociągowo-Kanalizacyjna Sp. z o.o., and operated by Spółka Katowickie Wodociągi S.A. Sewage sludge after dehydration in centrifuges contained 19.36% of dry matter (DM). The samples taken were analysed for heavy metal content and the percentage of organic matter, which in the case of the tested sediments constituted over 64% DM. The concentration of heavy metals according Polish regulations on sewage sludge based fertilizing productsdoes not exceed the permissible values^50^. All other parameters which characterize sewage sludge are contained in Table A2. The physicochemical composition was examined in an accredited GIG laboratory. The contents of Cd, Cr, Cu, Mn, Ni, Pb, Zn were examined by inductively coupled plasma atomic emission spectrometry (ICP-OES), mercury content was determined by vapor generation atomic adsorption spectrometry (CV AAS) and amalgamation technique.

The values of concentrations of target compounds in sewage sludge used in the production of fertilizers were presented in Table A3. The extraction of five grams of dried sewage sludge with 10 mL of methanol for four hours on a horizontal shaker at 50 rpm was performed. Subsequently, the samples were centrifuged. The final extract (8 mL) was transferred to 200 mL volumetric flasks, followed by the deionized water. Then, solid phase extraction (SPE) was performed (details, see Paragraph 2.6). The extracts were analysed by applying the method described in Paragraph 2.5.

### Characteristics of sewage sludge processing

The components of fertilizers and their amount enable the researchers to produce organo-mineral fertilizers which meet Polish requirements. According to The Regulation of the Minister of Agriculture and Rural Development, the organo-mineral fertilizers should contain at least 20% of organic matter, expressed as dry solids (Commission of European Communities (EC) No. 1069/2009 and (EC) No. 1107/2009, 2016/0084 (COD). The content of nutrients cannot be less than: 1% of total nitrogen, 0.5% of total phosphorus and 1% of total potassium. The content of heavy metals in organo-mineral fertilizers cannot exceed 100 mg for Cr, 5 mg for Cd, 60 mg for Ni, 140 mg for Pb and 2 mg for Hg. Moreover, the final fertilizers made from sewage sludge must be devoid of live eggs of intestinal parasites *Ascaris* sp.* Trichuris* sp.,* Toxocara* sp., as well as of bacteria of the genus *Salmonella.*

Fertilizers were produced on the basis of two production technologies–Granulation Technology and Capsulation Technology. The main substrate for their production was the sewage sludge characterized in “Characterization of sewage sludge” section. Fertilizers 1 and 2were obtained as a result of dynamic backward mixing, the component homogenization and mechanical granulation (thereafter referred to as Granulation Technology), while fertilizers 3 and 4were obtained as a result of mixing, homogenization and capsulation by means of polymeric carriers (thereafter referred to as Capsulation Technology).

#### Granulation Technology

The technological process of producing fertilizer granules took place in two stages. In the first one mixing and granulation takes place, in the second one–drying and packaging and customizing patent No. 233754^51^.

The technological line of the first stage includes the following processes:

I. dewatering of sludge, which takes place on centrifuges (efficiency oscillating around 22% of dry matter),
II. preparation of the feed on the mixer which consists in the homogenization with calcium oxide dolomite flour, gypsum and cellulose fibres,
III. redirecting it to the granulator.

Granules with a dry matter content of about 40–45% are formed as a result of the granulation process.

In the second stage of the technological process, granules were obtained as a result of drying at an ambient temperature of 20–24 °C. The drying process took 48 h. They [i.e. the granules] reach a dry matter content of granules of approx. 90% (permissible error limit ± 5%). The granulometric distribution of the final product was 2–6 mm.

In this way, fertilizers, which are indicated in the following tables as Fertilizer 1 and Fertilizer 2, were obtained (see Table A4).

#### Capsulation technology

In the first stage, sewage sludge was homogenized and mixed with a polymer carrier: (Fertilizer 3–2% sodium alginate and Fertilizer 4—sodium polyacrylate 0.1–1 mm granulation) drying and packaging and customizing patent No. 233754^52,53^.

In this way, fertilizers, which are indicated in the following Tables as Fertilizer 3 and Fertilizer4, were obtained (see Table A5).

The technological line of the first stage includes the following processes:

I. homogenization and mixing of sludge with polymers using an IKA homogenizer at a speed of 8.000 – 24.000 rpm
II. redirecting the sludge with a screw feeder to the capsulation line:
III. for Fertilizer 3: a technological line which consists of a reactor feeding the mixture to the tank with a cross-linking solution (calcium chloride concentration 2%);
IV. for Fertilizer 4: a technological line which consists of a stirred tank with a rotational speed of 100 rpm.

As a result of granulation, hydrated granules with a diameter of 3–6 mm are obtained.

In the second stage of the technological process, the obtained granules were dried at an ambient temperature of 20–24 °C up to a dry matter content of approx. 92% (permissible error limit ± 5%). The granulometric distribution of the final product was 2–5 mm.

### Leaching of selected components from the sewage sludge fertilizer

Leaching experiments were conducted as specified by the PN-EN 13,266 standard. 30 g of dried sewage sludge fertilizers were mixed with 1500 mL of deionized water, in dark glass bottles, weighed and blended for 24 h by means of a stirrer (300 rpm) at 25 ^0^C. After 24 h, the fertilizer suspensions were decanted. The supernatants were examined by means of the method described in Paragraph 2.5. Afterwards, fertilizers were mixed again with water to initial mass and mixed through 27 days. Then the fertilizer suspensions were decanted and the supernatants were prepared by solid-phase extraction (SPE) and they were analysed by means of the GC–MS/MS method, as described in Paragraph 2.5. The concentration of compounds in the solid phase (fertilizer) was calculated on the basis of the difference between the total and the water phase according to the following equation:

1 $$ C_{{{\text{solid}}}} = \, C_{{{\text{total}}}} - \, C_{{{\text{water}}}} $$

where C_solid_ is the concentration of compounds in the solid phase (fertilizer) in the equilibrium, C_total_ is the concentration in the fertilizer at the beginning of experiments, calculated on the basis of the contribution of compounds in sewage sludge, and C_water_ is the concentration in the water phase in the equilibrium. Concentration in the water phase in ng/L was converted to ng/g.

The desorption constant was calculated according to the following equation:

2 $$ K_{d} = \frac{{C_{solid} }}{{C_{water} }} $$

where C_solid_is the concentrations of compounds in the solid phase after a desorption experiment (expressed in ng/kg) and C_water_ (expressed in ng/L) is the concentration in the water phase.

### Environmental risk estimation

The risk quotations (RQ) were calculated for both fertilizers and soil, using the conventional methods for environmental risk assessment (ERA), on the basis of the measured concentrations of each pharmaceuticals in fertilizers and calculated predicted concentrations in soil and by comparing them with the predicted no effect concentrations (PNEC). ERA includes acute-and/or chronic toxicity data based on the most sensitive organism or combination of organisms within a given ecosystem to determine the PNEC of compound^54^. The Risk Quotient (RQ) values were calculated for each compound by dividing the measured or predicted environmental concentration (MEC/PEC) by a predicted no effect concentration (PNEC) by means of the following equation:

3 $$ RQ = \frac{MEC / PEC}{{PNEC}} $$

If the RQ values are below 0.1, no adverse effect is expected. If RQ values are between 0.1 and 1, the risk is low but the potential for adverse effects should be taken into consideration. If the RQ values are between 1.0 and 10, some adverse effect or moderate risk is probable. Finally, if the calculated RQ values are above 10, a high risk is anticipated^54,55^.

As recommended by the European Commission in the Technical Guidance Document on Risk Assessment EUR 20,418 EN/2 (EC-TGD 2003), the PEC_soil_ values were calculated according to the equation:

4 $$ PEC_{soil} = \frac{{C_{sludge} \times APPL_{sludge} }}{{DEPTH_{soil} \times RHO_{soil} }} $$

where C_sludge_ is the concentration of the pharmaceutical in fertilizer in µg/kg, APPL_sludge_ is the dry sludge application rate (0.5 kg/m^2^ year for agricultural soils), DEPTH_soil_ is the mixing depth of soil (0.2 m for agricultural soils) and RHO_soil_ is the bulk density of wet soil (1700 kg/m^3^ for agricultural soils).

PNEC_solid_ values were estimated from PNEC_water_ values by applying the equilibrium partition approach^56^:

5 $$ PNEC_{solid} = PNEC_{water} \times K_{d} $$

where K_d_ is the partitioning coefficient. Thevalues of K_d_ of fertilizers determined in this study were used to calculate the [amount]of the PNEC_fertilizer_. For the estimation of the RQ_soil_ of compounds in soil, PNEC_soil_ noted in the literature was used.

### Analytical procedures

For the isolation and enrichment of target compounds from sewage sludge fertilizer solvent extraction was performed. The clean-up step with SPE, the BAKER SPE 12G (J.T. Baker, Philipsburg, USA) system of the extracts was utilized. The evaporation of SPE extracts and derivatization were carried out with a thermo-block (AccuBlock Digital Dry Bath Labnet, Woodbridge, USA).

The samples (200 ml), spiked with 10 µL (10 µg/mL) of a mixture of internal standards were filtered with glass fibre filter (diameter of 45 mm; 0,4 µm) purchased from Macherey–Nagel. Samples were passed through the Oasis HLB 3 cc (60 mg / 3 mL) SPE cartridges (Waters), preconditioned sequentially with 2 mL of methanol and 2 mL of deionized water. Afterwards, the cartridges were dried and the analytes were eluted four times with 1 mL of methanol. The extracts (4 mL) were evaporated to dryness under a stream of argon using thermo-block. The silylation process with BSTFA + 1% TMCS was carried out at 65 ^0^C for 40 min in the thermo-block. The solutions were successively transferred into 2 mL chromatographic vials and analysed by GC–MS/MS.

The Thermo Scientific GC Trace 1300 gas chromatograph coupled with a ITQ 900 ion trap mass spectrometer and a TriPlus RSH autosampler was used to perform the analyses. The details of the analytical method described elsewhere^57^. Xcalibur software was used for the collection of data, and the analysis and processing of the said data.

The highest characteristic precursor ion/product ion transitions and characteristic ions that were used are listed in Table A6. Specific and intense product ions of each target analyte were used for quantification. The qualifier ion was a secondary product ion for the sake of confirmation.

Recoveries for each analyte for the method were determined by spiking the liquid (n = 5) and solid samples (n = 3) with a standard solution and tested according to the same methodology as that which was associated with leaching experiments (see Table A7).The volume of 200 mL of liquid samples were mixed with 100 µL of methanol stock standard solution to the final concentration in the 500 ng L^-1^sample. The solid samples (fertilizers), five grams, were mixed with 1 mL of standard solution to the final concentrations 600 ng g^-1^.

## Results and discussion

### The release of pharmaceuticals

**Figure 1:**
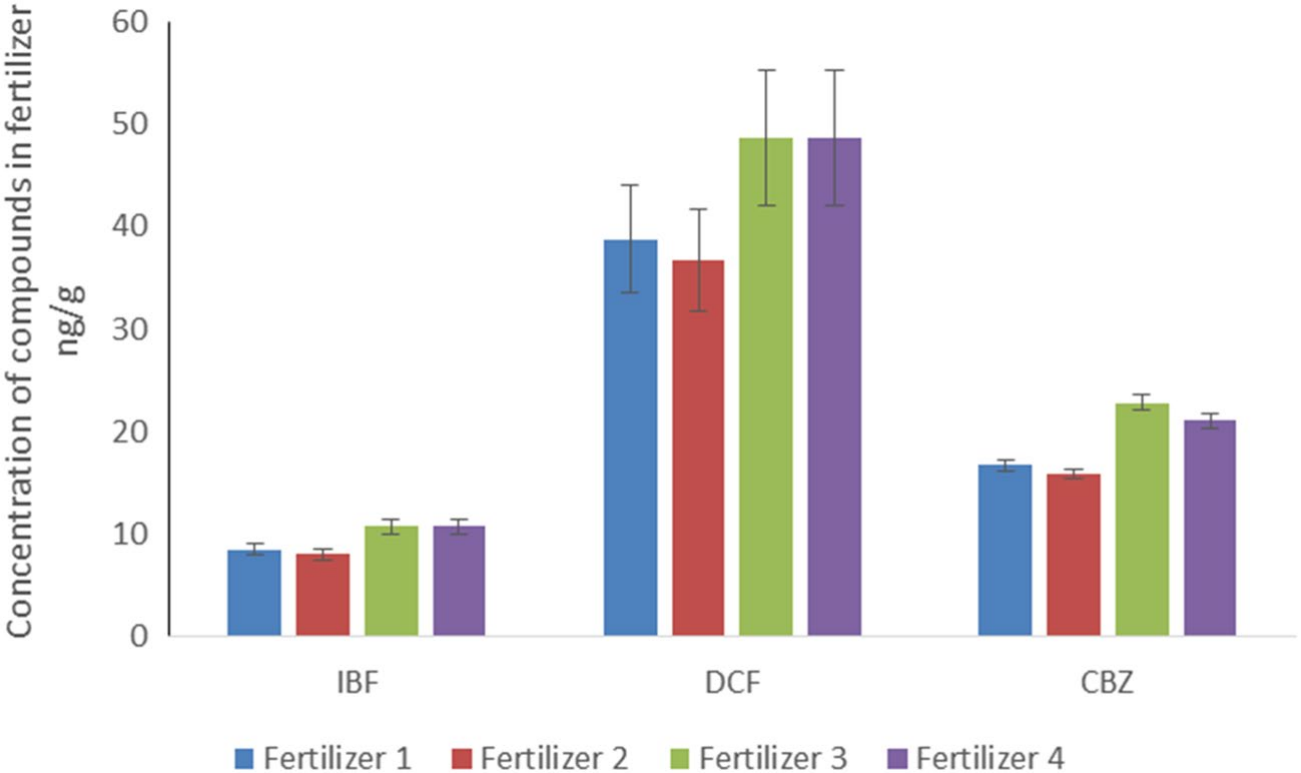
Concentrations of compounds in fertilizers expressed in ng/g.

Concentrations of target compounds in tested fertilizers were determined on the basis of their contribution in raw sewage sludge and its contribution in fertilizers (see Fig. 1 and Table A8).The differences in the content of individual pharmaceuticals in the tested fertilizers result from the amount of sewage sludge used for their production and the content of pharmaceutical residues in the sludge. For Fertilizer 1 and 2, sewage sludge constituted 70–74% of the initial product mass, and for Fertilizers 3 and 4 –95% of the product initial weight. As a result, the obtained fertilizers contained the most diclofenac per gram of the product, from 36 to 52 ng/g, and from 8 to 11 ng/g and 16 to 22 ng/g ibuprofen and carbamazepine, respectively (Fig. 1).

**Figure 2:**
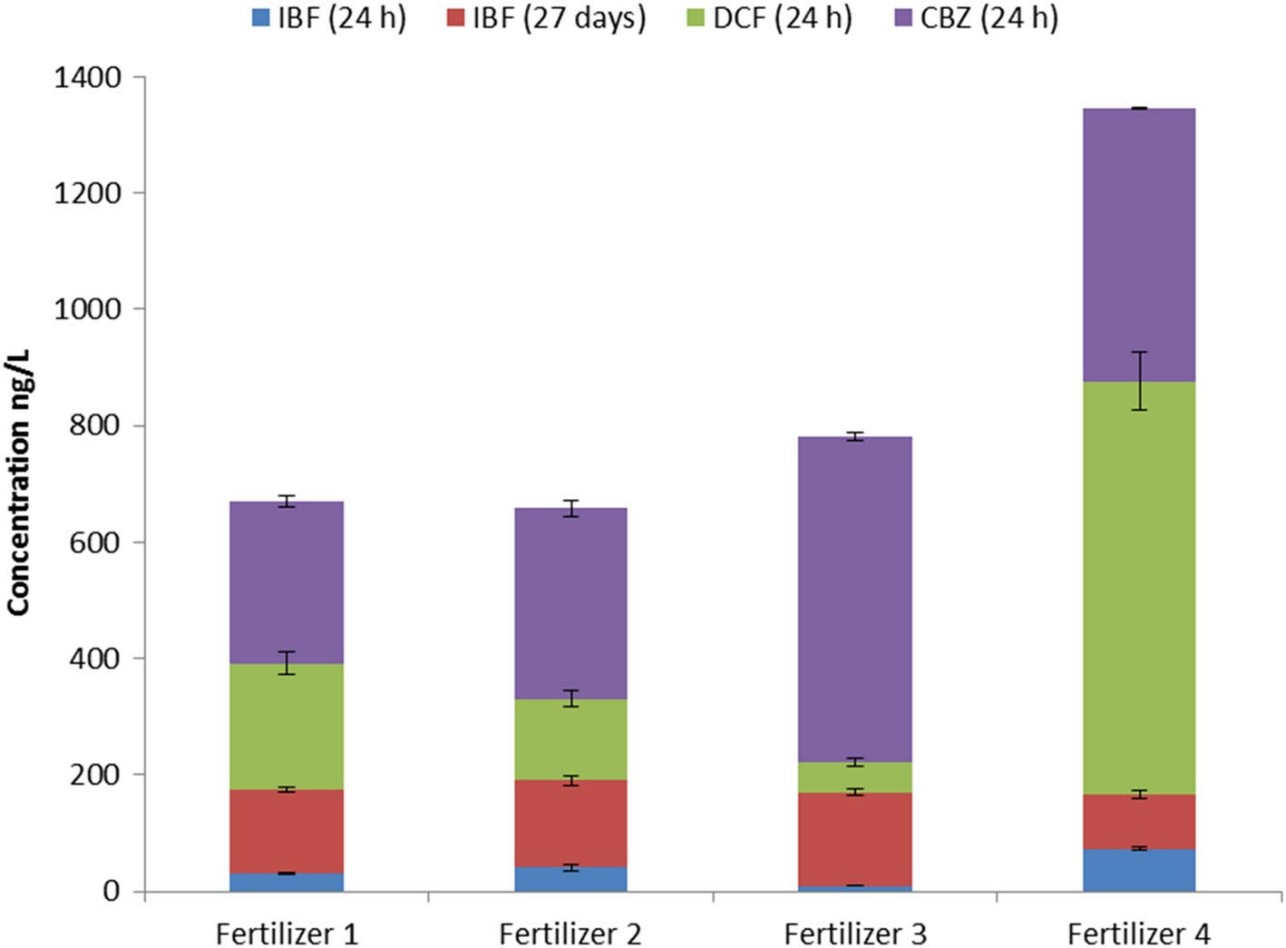
Average concentrations (ng/L) of pharmaceuticals in the leachate after 24 h and 27 days for tested fertilizers. Only ibuprofen was observed in the water phase in a detectable concentration, after 27 days of leaching.

The release of tested compounds except estrogens confirmed their presence in tested fertilizers. Of the 5 tested micropollutants, 3 leached in detectable concentrations after 24 h contact of fertilizers with water (see Fig. 1).Estrogenic compounds were not detected in any of the fertilizers that were tested, what was probably the consequence of their low contribution in raw sewage sludge. Although DCF and CBZ were detected in fertilizers, they were not present in leachate after 27 days, in this study. After 27 days of leaching, only ibuprofen was observed in the water phase in a detectable concentration. The pharmaceutical concentrations reached in the water phase ranged from 9 ng/L to 709 ng/L. Carbamazepine was present in all tested liquid samples, at higher concentration in comparison to other compounds, except Fertilizer4, where the highest concentration was observed for diclofenac. The highest concentrations of ibuprofen and diclofenac, after 24 h, were observed in the leachate from Fertilizer 4. The highest concentration for carbamazepine was observed for Fertilizer 3 (Fig. 2). Thus, it can be clearly demonstrated that the leaching of compounds to the water phase is a complex process, and that it is determined by water contact, partitioning, mobility of compounds through the material and the composition of fertilizers. The difference in final concentrations obtained for four tested fertilizers suggests the strong effect of the fertilizer matrix to the leaching. The concentrations dynamics of ibuprofen, diclofenac and carbamazepine are quite different. The concentrations of PPCPs in the fertilizers and after their land application can be reduced as a results of physical, chemical and biological processes. According to experiments of Tsekoura et al. concerning half-lives of PPCPs in aerobic dissipation, DCF was the fastest biodegradable compound (t_1/2_ = 7.2 days), while IBF was not removed to a percentage higher than 50% during the test (50 days)^58^. On the contrary, in the study of biodegradation reported by Xu et al., the half-life of IBF in soil ranged from 0.91 to 6.09 days^59^. In another study of Xu et al. the half-life of IBF was 7.2 days.

### Equilibrium partitioning

**Figure 3:**
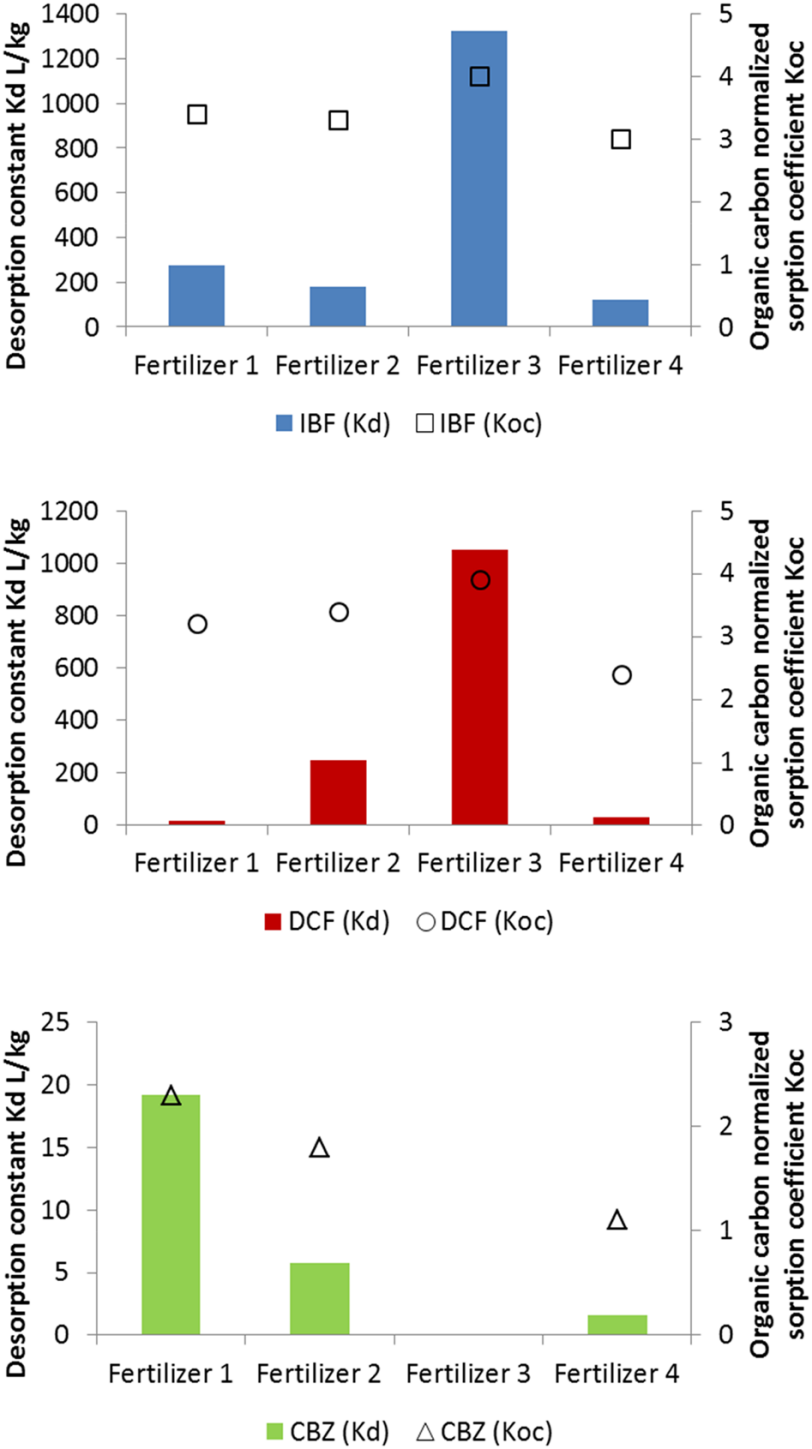
Comparison of the desorption constant (K_d_) in L/kg and organic carbon normalized sorption coefficient K_OC_ of pharmaceuticals between fertilizers obtained during the time equilibrium, 24 h contact time with water. The fraction of organic carbon (f_OC_) in kg/kg were calculated on the basis of the amount of raw sewage sludge used in the production of fertilizer and contribution of OC in the sludge.

The desorption constant values given in Fig. 2 were calculated according to Eq. (2). The value of K_d_ for CBZ and Fertilizer 3, was not measurable because all compound was present in the water phase. For the remaining fertilizers, almost the entire amount of CBZ was present in water phase, which signifies the lowest values of K_d_ observed for carbamazepine. Much higher values of K_d_ were noted for IBF and DCF. The occurrence of ibuprofen, diclofenac and carbamazepine, mainly in ionic form in the natural pH of fertilizer, could be related with their desorption to the water phase. However, DCF was not observed in the leachate after 27 days. One of the key factors affecting the transport of PPCPs in soils is their dissipation through degradation. This is associated either with an accumulation and/or (bio)degradation of these compounds, thus it causes low mobility in the soil. A similar effect in reference to CBZ, DCF and IBF mobility was observed by Lachassagne et al.^60^. According to the latter, the distribution of carbamazepine and ibuprofen was not influenced by liming and anaerobic digestion. Both [substances] were predominantly present in the soluble fraction. The aqueous solubility and relatively low log K_OW_ of carbamazepine indicate its low potential to sorption onto a particulate matrix^61^. The sorption potential of hydrophobic contaminants could be estimated according to their n-octanol/water partitioning coefficient (K_OW_). Contaminants with log K_OW_ < 2.5 would have low sorption potential, those with log K_OW_ between 2.5 and 4 would have a medium sorption potential and those with log K_OW_ higher than 4 would have high sorption potential. However, it should be noted that the type of the solid matrix and other interactions such as electrostatic interactions, cationic exchanges play an important role in sorption^62–64^. The resulting partitioning coefficient values normalized on organic carbon content (log K_OC_) are indicated in Fig. 3.The mobility of micropollutants in the solid phase depends on chemical and physical properties of the chemicals. Pharmaceuticals with acidic chemical properties, such as NSAIDs, show medium to high mobility, which are also reflected in this study. The pharmaceuticals with neutral and basic chemical properties are retained in greater amounts by the solid phase, such as soil and sewage sludge. Pharmaceuticals with neutral chemical properties are generally more hydrophobic than charged spices (Table A1) and partition to a greater extent to the organic content in the soil. The octanol–water partition coefficient K_OW_ was used to represent hydrophobicity. However, the sorption studies of organic compounds in various solutions cannot be interpreted by one or two mechanisms. The higher release of carbamazepine could be the result of its higher contribution in fertilizers in comparison to NSAIDs. The lower value of K_OW_ of carbamazepine in comparison to ibuprofen and diclofenac suggested its higher mobility. Moreover, the pharmaceuticals with cationic chemical properties are repelled by the positive charged surface of solid particles. As partially presented above, the mobility of selected pharmaceuticals to the water phase was moderately dependent on the organic carbon content and contribution of ionic and/or hydrophobic interaction between solid particles and molecules of compounds.

The values of log K_OC_ for the tested system varies between 1.1 (carbamazepine) and 4.0 (ibuprofen). The values of log K_OC_ obtained for compounds suggest a low sorption potential of CBZ (1.1–2.3) and a medium sorption potential of IBF (3.0–4.0) and DCF (2.4–3.9). The results of log K_OC_ of CBZ and IBF match the log K_OW_. Diclofenac has high values of log K_OW_ (4.2–4.5), but it manifested markedly lower sorption potential to organic carbon. Considering the composition of fertilizers, the highest sorption potential of IBF and DCF was observed for Fertilizer 3, with sodium alginate as a binder. The highest desorption of compounds was observed for Fertilizer 4, where acrylate is used as a binder. A similar behaviour of biocide in the acrylate render was observed for biocides^65^. More mineral products (Fertilizers 1 and 2) manifested a better sorption capability for CBZ, which could suggest electrostatic interactions effects on its sorption. An electrochemical affinity connected with the existence of aromatic rings and electron-donating and/or accepting functional groups in the compounds and fertilizers also play a significant role.

### Mass balance of pharmaceuticals

**Figure 4:**
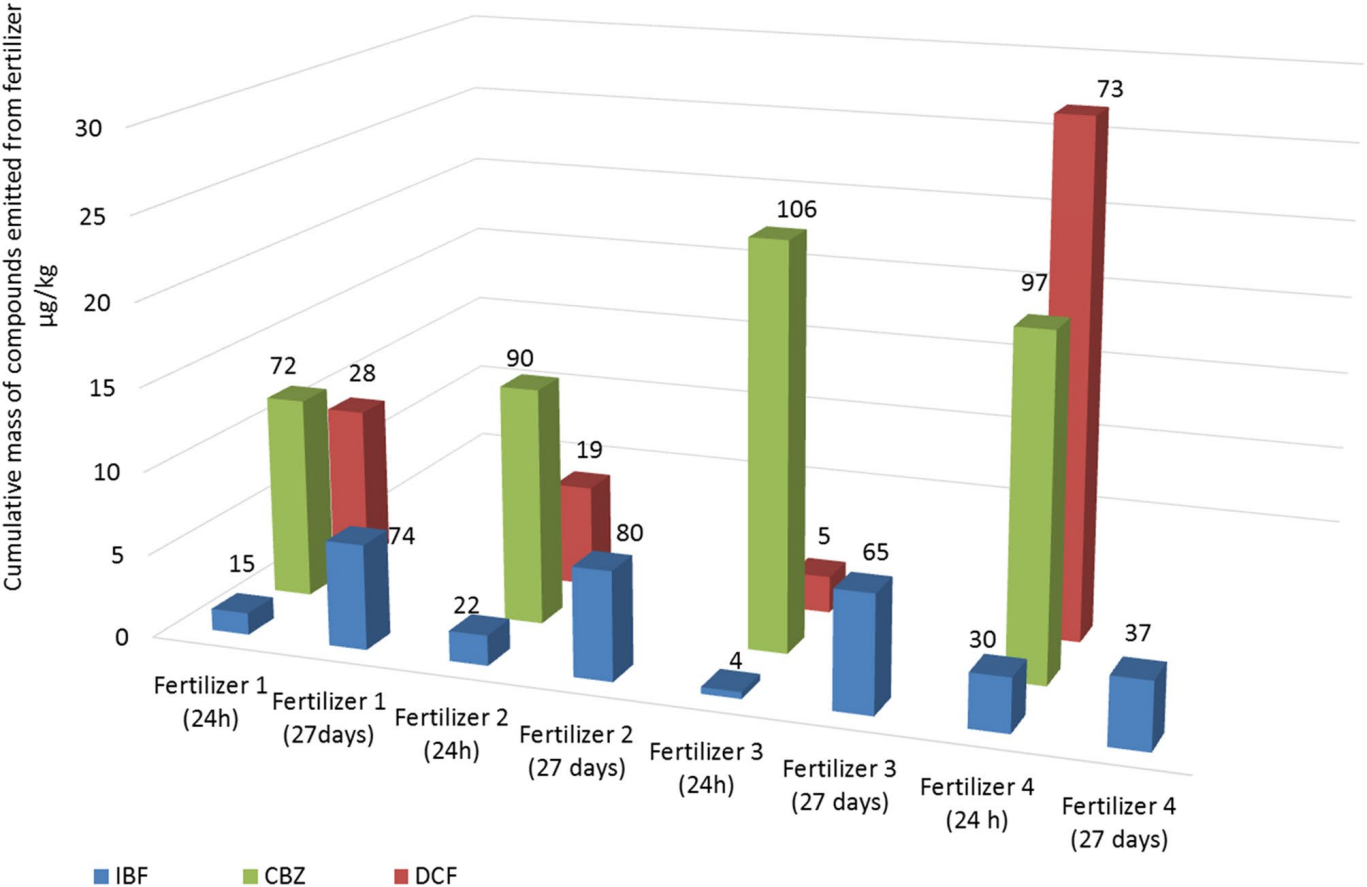
Cumulative mass of pharmaceuticals per unit mass (µg/kg) emitted from fertilizers in dependence of different wetting times at room temperature (22 °C). The values represented by the columns above are the weight percentage of initial mass of pharmaceuticals leached from the respective fertilizers during the experiments. The values of the weight percentage which exceed 100% are the result of uncertain as homogenization of raw sewage sludge and the drying process of the product.

By calculating the leached fraction of pharmaceuticals during a period of 24 h one observed that less than 10% of IBF and DCF were leached from Fertilizer 3 (see Fig. 4). While for IBF and DCF a significant fraction—over 15%, for CBZ—over 70% –of the total mass was leached for the remaining fertilizers. A much higher fraction of IBF (37–80%) was leached after 27 days of water contact for each fertilizer. However, it can be clearly stated that in this mass balance assessment, water contact in a period of 24 h leads to the highest emission of CBZ, except for Fertilizer 4, where DCF also has a high emission. Among NSAIDs, higher emission at 24 h was noted for diclofenac. The total emission of IBF after 28 days was considerably higher for all fertilizers, which indicates that the emission associated with soaked materials should the greatest.

The obtained results are consistent with the results (Lachassagne et al., 2015), who showed the greatest potential of ibuprofen desorption from sewage sludge after spreading them on the ground^60^. Similarly, diclofenac was present in the leachate taken from the soil in this study. In spite of the fact that the presence of carbamazepine in sewage sludge was confirmed, its presence in soil leachate has not been detected. The authors point to the potential for carbamazepine accumulation and low mobility of this compound from soil.

### Environmental risk

Pharmaceuticals residues transported with biosolids to soils can represent a potential risk to the whole ecosystem because of their mobility. Table 1 shows ecotoxicological data of pharmaceuticals and their RQ values obtained for fertilizers. The RQ values for soils are not presented because the results of calculations were much below 0.01 for all tested compounds.

The compounds responsible for the low but the potentially adverse effects were CBZ and DCF. The RQ values were 0.2 and 0.93 for CBZ in Fertilizers 2 and 4 and 0.17 for DCF in Fertilizer 4. RQ values of IBF were much lower than 0.1. Generally, no risk is expected to be involved in reference to the fertilizers and soil amended with these biosolids. The results of research work demonstrated that sewage sludge is a source of raw materials, useful for the production of fertilizers. They are in line with other works^60,66–69^. However, the continuation of research in terms of the monitoring of organic contaminants mobility and transformation is necessary.

Chemical analyses have shown that the obtained fertilizers meet Polish requirements, the concentration of heavy metals does not exceed required values and no trace of parasites and pathogenic bacteria were observed. In this respect, the tested fertilisers did not pose an environmental hazard.

## Conclusions

The results of the study confirmed the release of pharmaceuticals from the fertilizers, (less than from pure sewage sludge). However, the process depends on the type of the technology of fertilizer production that is used.

As a result of the study, differences in the leaching of micro-impurities to methanol between unprocessed sludge and fertilizer products based on sewage sludge were observed. A decrease in the leaching of ibuprofen and diclofenac was observed. It should be borne in mind that fertilizer products prepared for testing were not subjected to high-temperature drying processes. The analysed materials dried at ambient temperature, hence it is justified to continue research in the field of processing sewage sludge into environmentally safe fertilizer products. Limiting the leaching of pharmaceutical compounds and maximizing their decomposition at the production stage is important from the point of view of processing sewage sludge into fertilizer products. As could be seen in water extracts after 27 days, the majority of substances except ibuprofen were practically undetectable. In view of the above, the important conclusion is that this compound is difficult to decompose and leaches from sewage sludge over a long period of time.

It should be stressed that the introduction of ibuprofen into the environment together with fertilizer products based on sewage sludge may cause a risk for the environment. Therefore, in spite of the lack of legal regulations, attempts should be made to carry out further research into the spreading of pharmaceuticals into the environment via waste products from WWTPs and to introduce permanent monitoring of the presence of pharmaceuticals in soils fertilized with these products.

## Supplementary Information

Supplementary Information.

## Data Availability

All data generated or analysed during this study are included in this published article and its supplementary information files.
