# Supplementary material for: The impact of sewage sludge processing on the safety of its use
Source: Sci Rep. 2022 Jul 18;12:12227. doi: 10.1038/s41598-022-16354-5 (PMC9293921; doi:10.1038/s41598-022-16354-5)
Supplement: Supplementary file 1 — Supplementary Information. [file 41598_2022_16354_MOESM1_ESM.docx]

**Supplementary materials**

**The impact of sewage sludge processing on the safety of its use**

Katarzyna Styszko^1*^, Justyna Durak^2^, Beata Kończak^3^, Marcin Głodniok^3^, Anna Borgulat^3^

^1^ AGH University of Science and Technology, Faculty of Energy and Fuels, Department of Coal Chemistry and Environmental Sciences, Al. Mickiewicza 30, 30-059 Krakow, Poland

^2^ Cracow University of Technology, Faculty of Environmental and Power Engineering, Department of Geoengineering and Water Management, Krakow, Poland

^3^ Central Mining Institute, Department of Water Protection, Plac Gwarków 1, 40-166 Katowice, Poland

*corresponding author: K. Styszko, styszko@agh.edu.pl

**Table A1.** Chosen PPCPs and their properties

| Group | Compound | CAS No | Molecular formula | MW | pK_a_ | Log K_OW_ |
| --- | --- | --- | --- | --- | --- | --- |
| Anti-inflammatory/analgesics | Ibuprofen (IBF) | 15687-21-1 | C_13_H_18_O_2_ | 206,28 | 4.9 | 3.5-4.0 |
|  | Diclofenac (DCF) | 15307-79-6 | C_14_H_10_Cl_2_NNaO_2_ | 318.10 | 4.2 | 4.2-4.5 |
| Antiepileptic drug | Carbamazepine (CBZ) | 298-46-4 | C_15_H_12_N_2_O | 236.27 | 13.9 | 2.4-2.9 |
| Estrogens | β-Estradiol (E2) | 50-28-2 | C_18_H_24_O_2_ | 272.39 | 10.3 | 4.0 |
|  | 17α-Ethinylestradiol (EE2) | 57-63-6 | C_20_H_24_O_2_ | 296.41 | 10.3 | 3.4 |
| Internal standards | Ibuprofen-D_3_ | 121662-14-4 | C_13_D_3_H_15_O_2_ | 209,30 | - | - |
|  | Diclofenac-^13^C_6_ | 1261393-73-0 | ^13^C_6_C_8_H_10_Cl_2_NNaO_2_ | 405.16 | - | - |
|  | 17β-Estradiol-D_5_ | 221093-45-4 | C_18_H_19_O_2_D_5_ | 277.41 | - | - |

**Table A2.** Characterization of sewage sludge

| Component | Content / Determined value |
| --- | --- |
| total moisture, wt. % (% m / m) | 80.64 |
| dry matter,% wt. (% m / m) | 19.36 |
| organic substance, wt.% DM | 64.61 |
| volatile matter, wt. DM | 54.96 |
| ash, wt.% DM | 35.39 |
| carbon, wt.% DM | 34.52 |
| hydrogen, wt. DM | 4.98 |
| nitrogen, wt.% DM | 8.80 |
| oxygen, wt.% DM | 15.16 |
| total sulphur, wt. DM | 1.20 |
| ash sulphur, wt. DM | 0.05 |
| combustible sulphur, wt. DM | 1.15 |
| phosphorus, wt. DM | 3.68 |
| potassium, wt. DM | 0.50 |
| magnesium, wt. DM | 0.92 |
| calcium, wt.% DM | 2.34 |
| Iron, wt.% DM | 5.23 |

**Table A3.** Concentrations of selected compounds in sewage sludge used as a substrate to production of fertilizer

| Compound | Concentration  ng/g |
| --- | --- |
| IBF | 53.6 ± 3.5 |
| DCF | 243.6 ± 32.9 |
| CBZ | 101.9 ± 3.3 |
| E2 | <LOQ |
| EE2 | <LOQ |

**Table A4**. The composition of fertilizers 1 and 2 based on sewage sludge obtained in granulation technology

| Composition | Fertilizer 1 | Fertilizer 2 |
| --- | --- | --- |
|  | Percentage share (by weight) | |
| Sewage sludge | 74 | 70 |
| Burnt lime | 4 | 19 |
| Dolomite flour | 20 | x |
| Gypsum | x | 10 |
| Cellulose fibres | 1 | 1 |

**Table A5.** The composition of fertilizers 3 and 4 based on sewage sludge obtained in capsulation technology

| Composition | Fertilizer 3 | Fertilizer 4 |
| --- | --- | --- |
|  | Percentage share (by weight) | |
| Sewage sludge | 95 | 95 |
| Sodium Alginate | 5 | x |
| Sodium polyacrylate | x | 5 |

**Table A6.** Selected parameters of method: retention times, MS/MS parameters, method quantitation limits for the analysis of the chosen PPCPs by GC-MS/MS

| Compound | RT  min | Precursor-ion m/z | Product-ions  Quantification/  confirmation m/z | Internal standard | Linearity range  ng/mL | R^2^ | MQL  ng/g  Solid  sample | MQL  ng/L  Liquid sample |
| --- | --- | --- | --- | --- | --- | --- | --- | --- |
| IBF | 9.73 | 160 | 145/117 | Ibuprofen-D_3_ | 2-2500 | 0.9909 | 4.51 | 3.50 |
| DCF | 14.38 | 214 | 179/151 | Diclofenac-^13^C_6_ | 2-2500 | 0.9903 | 6.61 | 4.42 |
| CBZ | 13.72 | 193 | 165/191 | Carbamazepine-D_10_ | 10-2500 | 0.9914 | 21.23 | 20.00 |
| E2 | 14.99 | 416 | 285/326 | β-Estradiol-D_5_ | 5-2500 | 0.9930 | 9.97 | 10.15 |
| EE2 | 15.60 | 425 | 409/393 | β-Estradiol-D_5_ | 5-2500 | 0.9901 | 14.54 | 13.78 |

**Table A7.** Recoveries of target compounds

| Compound | Absolute Recovery %  Solid sample | Relative Recovery %  Solid sample | Absolute Recovery %  Liquid sample | Relative Recovery %  Liquid sample |
| --- | --- | --- | --- | --- |
| IBF | 48.5 | 87.6 | 61.8 | 94.4 |
| DCF | 40.3 | 69.7 | 73.4 | 100.9 |
| CBZ | 32.4 | 89.1 | 34.4 | 78.6 |
| E2 | 50.4 | 96.6 | 49.5 | 103.5 |
| EE2 | 52.4 | 89.7 | 55.3 | 85.5 |

**Table A8.** Concentrations (ng/g) with standard deviation of compounds in fertilizers

| Compound | Fertilizer 1 | Fertilizer 2 | Fertilizer 3 | Fertilizer 4 |
| --- | --- | --- | --- | --- |
| IBF | 8.5 ± 0.6 | 8,1 ± 0.5 | 11.6 ± 0.8 | 10.7 ± 0.7 |
| DCF | 38.8 ± 5.2 | 36.7 ± 5.0 | 52.7 ± 7.1 | 48.7 ± 6.6 |
| CBZ | 16.8 ± 0.5 | 15.9 ± 0.5 | 22.8 ± 0.7 | 21.1 ± 0.7 |
